# The Bologna motor and non-motor prospective study on parkinsonism at onset (BoProPark): study design and population

**DOI:** 10.1007/s10072-020-04305-9

**Published:** 2020-03-26

**Authors:** Giovanna Calandra-Buonaura, Luisa Sambati, Francesca Baschieri, Maria Vitiello, Manuela Contin, Caterina Tonon, Sabina Capellari, Federica Provini, Pietro Cortelli, on behalf of the BoProPark Study Group, Giorgio Barletta, Giuseppe Caltabiano, Annagrazia Cecere, Roberto Gallassi, Giulia Giannini, Pietro Guaraldi, Raffaele Lodi, Giovanna Lopane, David Neil Manners, Paolo Martinelli, Filomena Miele, Francesco Mignani, Susan Mohamed, Stefania Nassetti, Federico Oppi, Piero Parchi, Giulia Pierangeli, Roberto Poda, Cesa Scaglione, Laura Solieri, Michelangelo Stanzani-Maserati, Claudia Testa

**Affiliations:** 1 IRCCS Istituto delle Scienze Neurologiche di Bologna, Via Altura 3, 40139 Bologna, Italy; 2 Dipartimento di Scienze Biomediche e Neuromotorie, Università di Bologna, Bologna, Italy; 3 Diagnostica Funzionale Neuroradiologica, IRCCS Istituto delle Scienze Neurologiche di Bologna, Bologna, Italy

**Keywords:** Parkinson’s disease, Atypical parkinsonisms, Cohort study, Study design, Motor symptoms, Non-motor symptoms

## Abstract

**Objective:**

The Bologna motor and non-motor prospective study on parkinsonism at onset (BoProPark) was designed to prospectively characterize motor and non-motor features in patients with a progressive neurodegenerative disease starting with parkinsonism since early disease stage and to investigate their diagnostic and prognostic role in the differential diagnosis of Parkinson’s disease from atypical parkinsonisms. The aim of this paper is to describe the method and population of the BoProPark study.

**Methods:**

Patients referred to our Department with parkinsonism within 3 years from motor onset were recruited. Secondary causes of parkinsonism were excluded. Each patient underwent a comprehensive evaluation of motor and non-motor symptoms, assessed by means of quantitative, objective instrumental tests in addition to scales and questionnaires. The evaluations were performed at enrolment (T0), after 16 months (T1) and after 5 years (T2). Diagnoses were made according to consensus criteria.

**Results:**

We recruited 150 patients, with mean age 61.5 ± 9.8 years and mean disease duration 20 ± 9 months. H&Y stage was 1 in 47.3% and 2 in 46.7% of cases. Mean UPDRS-III was 17.7 ± 9.2. Fifty-four patients were on dopaminergic treatment with median levodopa equivalent daily dose (LEDD) of 200 mg.

**Conclusions:**

We expect that the prospective nature of the BoProPark study as well as the comprehensive, instrumental evaluation of motor and non-motor symptoms in patients with parkinsonism will provide important new insights for both clinical practice and research. Our data could be used for comparison with other cohorts and shared with national and international collaborators to develop new innovative projects.

## Introduction

Parkinsonism is defined by the presence of a combination of bradykinesia, rigidity, tremor, and postural instability. It may be due to secondary causes (e.g., vascular encephalopathy, drugs’ side effects) or may occur in the context of neurodegenerative disorders, such as Parkinson’s disease (PD) or atypical parkinsonisms (APs). PD is the second most common neurodegenerative disease, carrying a high burden in terms of disability and healthcare cost worldwide [1]. APs are distinct entities, which at early disease stage could mimic PD [2], but during the disease course develop motor or non-motor features that exclude or are atypical for PD. Moreover, APs are characterized by a worse prognosis and different therapeutic needs compared to PD.

Nowadays, with the better understanding of the pathogenesis of these conditions and the aim of the development of disease-modifying therapies [3–5], the need for finding suitable clinical and instrumental markers to confirm diagnosis and track progression has become of crucial importance. Several prospective studies have been promoted for this purpose, focusing on frequency, severity, impact on diagnosis, and prognosis of motor as well as non-motor symptoms (e.g., autonomic failure, sleep disorders, cognitive impairment). However, most of these studies mainly assessed non-motor symptoms by means of questionnaires and scales that, while being comprehensive and standardized screening tools, do not allow objective quantification and only capture patient self-reported symptoms [6]. Moreover, the majority of these studies targeted patients with a single diagnosis, even though differentiating PD from APs at onset is still challenging [7].

The Bologna motor and non-motor prospective study on parkinsonism at onset (BoProPark) was designed to prospectively characterize motor and non-motor features, assessed by means of quantitative, objective instrumental tests in addition to scales and questionnaires, in patients with a progressive neurodegenerative disease starting with parkinsonism since early disease stage and to investigate their diagnostic and prognostic role in the differential diagnosis of these diseases.

The aim of this paper is to describe the method and population of the BoProPark study.

## Materials and methods

The BoProPark is a prospective, observational, single-center study.

We enrolled all consecutive patients aged 18–80 years presenting with parkinsonism (tremor, bradykinesia, rigidity, postural instability) with a progressive course and within 3 years from motor onset that were referred from September 2007 up to November 2018 to the Movement Disorders Clinic of the Department of Biomedical and Neuromotor Sciences, University of Bologna. The neurodegenerative origin of the parkinsonism was confirmed in all patients by pathological single-photon emission computerized tomography for imaging the dopamine transporter (SPECT DaTSCAN). Secondary causes of parkinsonism were excluded before enrollment by means of appropriate investigations including brain magnetic resonance imaging (MRI). Other exclusion criteria to enter the study were concurrent clinically severe medical or psychiatric disease that could have interfered with study results.

The study was initially supported by the strategic research program “Bando ricerca finalizzata 2006” of the Italian Ministry of Health (reference number RFPS2006-7-336374) and subsequently continued as independent research and approved by the Ethics Committee of the Local Health Authority of Bologna (reference number 09070). The study was performed in accordance with the Declaration of Helsinki.

All participants gave their written informed consent to participate in the study.

### BoProPark protocol

According to the BoProPark study, each patient underwent the same protocol (Table 1) at baseline (T0), after 16 months (T1) and 5 years (T2). The time window between T0 and T1 evaluations was chosen in order to get information on the early evolution of the disease, i.e., within the first 5 years, when correct clinical diagnosis is more challenging [29]. The second follow-up (T2) was set at reasonable time to collect data on an established clinical diagnosis, considering that PD and APs have different progression and prognosis. Moreover, because inclusion criteria requires that patients have parkinsonism within 3 years from motor onset, this time frame allowed us to evaluate most of the APs over their disease course, taking into account that survival in this cases is usually less than 10 years.

**Table 1:** The BoProPark protocol

General clinical assessment	-History taking -Complete neurological examination
Motor assessment	-Unified PD Rating Scale - part III [8] -Hoehn & Yahr stage [9] -Subacute challenge test with levodopa [10]
Autonomic system	-Cardiovascular reflex tests [11, 12] -Scales for Outcomes in Parkinson’s Disease – Autonomic questionnaire [13]
Sleep	-Whole-night video-polysomnography [14] -Parkinson’s disease sleep scale 1 [15] -REM sleep behavior disorder questionnaire [16] -Bologna questionnaire on sleepiness-related symptoms [17] -Restless Legs Syndrome criteria according to the International Classification of Sleep Disorders [18] and International Restless Legs Syndrome Study Group rating scale [19, 20] -Epworth sleepiness scale [21]
Quality of life	-PD quality of life questionnaire [22]
Cognitive and behavioral	-Mini-Mental State Examination [23] -Simple Copy Design Test [24] -Selective Visual Attention Test (Stroop Test) [25] -Phonemic and Semantic Verbal Fluency Test [26] -Brief Mental Deterioration Battery [24] -Beck’s Depression Inventory [27] -State-Trait Anxiety Inventory [28]

Drug naive patients at T0 started levodopa plus dopa-decarboxylase inhibitor (carbidopa or benserazide) treatment titrated in 1 month up to 200 + 50 mg/die.

The protocol included the following evaluations:

- History taking: age, sex, occupation, family history, past medical history, drug history and concomitant medications, age at disease (parkinsonism) onset, disease duration, characteristics of onset and progression, symptoms at initial presentation, occurrence of motor and non-motor symptoms (history consistent with bradykinesia, rigidity, tremor, postural instability, other additional movement disorders such as dystonia or myoclonus and their body distribution, falls, dysphagia, dysarthria, dysphonia, higher mental functions, depression, sleep disturbances, autonomic symptoms including symptoms suggestive of orthostatic hypotension, urinary urgency, frequency, incontinence, incomplete bladder emptying, sexual dysfunction, sweating abnormalities, constipation, diarrhea, vision disturbances); severity, timing and latency from disease onset were recorded for each symptom and sign; dopaminergic treatment.
- Body mass index.
- Neurological examination.
- Quantification of motor impairment and disease severity by means of Unified PD Rating Scale - part III (UPDRS-III) and Hoehn & Yahr (H&Y) stage [8, 9].
- Patients treated with levodopa underwent quantification of motor response to levodopa through a subacute challenge test with a standard oral dose of levodopa plus carbidopa or benserazide (100 + 25 mg) based on a kinetic-dynamic approach [10]. Drug naïve patients at T0 underwent this examination within 6 months after the end of levodopa titration. Patient’s motor response was objectively assessed before and after standard intervals from drug dose with finger tapping performed on a computer-based system; simultaneous blood venous samples were withdrawn for measuring levodopa plasma concentration; dyskinesias, when present, were also rated at the same times as motor responses by the Clinical Dyskinesia Rating Scale [30]; blood pressure in supine position and after 3 min of standing was measured before and 1 h after drug administration to detect possible levodopa-induced or worsened orthostatic hypotension.
- Evaluation of autonomic control of the cardiovascular system through cardiovascular reflex tests performed according to standardized procedures with continuous monitoring of beat-to-beat blood pressure, heart rate, oronasal airflow, abdominal breathing, and peripheral vasomotor tone and video: tilt test (65° for 10 min), Valsalva’s maneuver (forced expiratory pressure of 40 mmHg for 15 s), deep breathing test (6 breaths/min), cold face test (cold stimulus on forehead for 1 min), handgrip test (1/3 of maximal effort for 5 min) [11, 12]; blood pressure and heart rate changes from baseline in response to these maneuvers were used to calculate indices of sympathetic and parasympathetic activity and baroreflex integrity and to detect the presence of orthostatic hypotension.
- Presence of symptoms of autonomic dysfunction through the questionnaire Scales for Outcomes in Parkinson’s Disease – Autonomic (SCOPA-Aut) [13].
- Sleep study by means of whole-night video-polysomnography: electroencephalogram (C3-A2, O2-A1, Cz-A1), right and left electrooculogram, surface electromyogram from submental, intercostalis, right and left extensor carpi radialis and tibialis anterior muscles, tracheal microphone, oronasal airflow, thoracic and abdominal respirogram, electrocardiogram, oxyhemoglobin saturation by means of finger oximeter and synchronized video recording [14].
- Sleep questionnaires and scales: PD sleep scale 1 [15], REM sleep behavior disorder (RBD) questionnaire [16], Bologna questionnaire on sleepiness-related symptoms [17], Restless Legs Syndrome criteria according to the International Classification of Sleep Disorders (ICSD-3) [18] and International Restless Legs Syndrome Study Group rating scale [19, 20], and Epworth sleepiness scale [21].
- Evaluation of quality of life by means of 39-item PD questionnaire [22].
- Cognitive and behavioral comprehensive assessment evaluating global cognition, verbal and visual memory, attention, executive and visuospatial function, language, depression, and anxiety. Neuropsychological evaluation included the following tests corrected for age, sex, and education according to Italian standardizations: Mini-Mental State Examination [23], Simple Copy Design Test [24], Selective Visual Attention Test (Stroop Test) [25], Phonemic and Semantic Verbal Fluency Test [26], and the Brief Mental Deterioration Battery, consisting of Rey’s Auditory Verbal Learning Test (immediate and delayed recall) [24], Visual Search Test (Barrage test) [24], Immediate Visual Memory Test [24], and Simple Verbal Analogies Test [31]. The Battery outcomes is a measure of global cognitive functioning, called Final Result [32, 33]. All patients also filled the 21-items Beck’s Depression Inventory [27] and the State-Trait Anxiety Inventory [28].

Demographic, clinical, and instrumental data were collected using a standard and anonymous form and entered in an ad hoc database. This database was specifically developed in Microsoft.net framework by SparkBio Srl (Bologna, Italy) for the purpose of collecting and storing data of this study in a comprehensive, standard, and safe way and making subsequent statistical analysis more straightforward. Moreover, this database allowed us to identify potential missing or erroneous information that were immediately pursued to guarantee the completeness and accuracy of the data. Importantly, the database was also implemented with a deterministic algorithm based on international criteria for PD [34], Parkinson’ disease with dementia (PDD) [35, 36], multiple system atrophy (MSA) [37], dementia with Lewy bodies (DLB) [38], progressive supranuclear palsy (PSP) [39], and corticobasal syndrome (CBS) [40] that checked the information and automatically provided a diagnosis that was recorded in the database itself and available for comparison and confirmation at future visits. In this regard, the database was designed as a questionnaire for all relevant diagnostic information needed to make a diagnosis according to the aforementioned criteria. Patients not fulfilling any diagnostic criteria were diagnosed as unspecified atypical parkinsonism (uAP). All diagnoses were independently confirmed by three neurologists expert in movement disorders who were blinded to the diagnosis provided by the database. Diagnoses made at baseline (T0) were revisited at T1 and T2 based on the results obtained from these follow-up evaluations.

Results of tests and questionnaires were recorded in ad hoc databases for statistical analysis.

Patients were also followed up at our department according to clinical practice and were regularly assessed for routine visits every 6–12 months as needed.

### Statistical analysis

In the present article, we performed a descriptive analysis using IBM SPSS Statistics software (version 25). The distribution of continuous variables was confirmed by visual analysis of the Q-Q plots and the Shapiro Wilk test. Continuous variables were expressed using mean and standard deviation while categorical variables using frequencies and proportions.

## Results

**Fig. 1:**
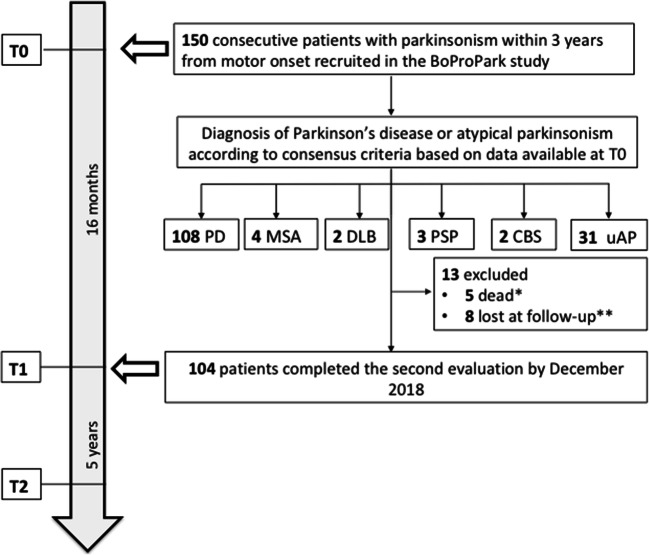
Study flowchart, PD = Parkinson’s disease; MSA = multiple system atrophy; DLB = dementia with Lewy bodies; PSP = progressive supranuclear palsy; CBS = corticobasal syndrome; uAP = unspecified atypical parkinsonism. (*) all uAP (according to data available). (**) 6 PD and 2 uAP (according to data available)

A total of 150 patients were recruited. Demographics and clinical characteristics at T0 are reported in Table 2. Mean age was 61.5 ± 9.8 years, and mean disease duration was 20 ± 9 months. H&Y stage was 1 in 47.3% and 2 in 46.7% of cases. Mean UPDRS III was 17.7 ± 9.2. Fifty-four patients were on dopaminergic treatment with median levodopa equivalent daily dose (LEDD) of 200 mg (36–700). The following diagnoses were made at T0: 108 PD, 4 MSA, 2 DLB, 3 PSP, 2 CBS, and 31 uAP **(**Fig. 1**)**.

**Table 2:** Demographics and clinical characteristics of patients at T0

*N*	150
Males	78 (52%)
Age *(years)*	61.5 ± 9.8 (37–83)
Disease duration *(months)*	20 ± 9 (2–36)
Hoehn & Yahr stage	
1 2 3 and 4	71 (47.3%) 70 (46.7%) 9 (6%)
UPDRS-III	17.7 ± 9.2 (5–51)
LEDD (mg)*	200 (36–700)
Diagnosis	
PD MSA DLB PSP CBS uAP	108 (72%) 4 (2.7%) 2 (1.3%) 3 (2%) 2 (1.3%) 31 (20.7%)

Data are expressed as number (percentage) or mean ± standard deviation (min – max)

*Data expressed as median (min – max)

## Discussion

The BoProPark study aims to describe and evaluate the diagnostic and prognostic role of motor and non-motor symptoms in a cohort of patients with a neurodegenerative disease starting with parkinsonism within 3 years from motor onset and prospectively followed up to reach a more robust clinical diagnosis. Motor and non-motor symptoms were evaluated by means of clinical and instrumental assessments according to standardized procedures. According to the results of clinical and instrumental assessments, diagnoses were revisited and confirmed at each evaluation (T0, T1, and T2) and at further follow-up.

In this paper, we describe study method and characteristics of patients at enrollment. Our cohort is representative of parkinsonian patients at disease onset referring to a tertiary Movement Disorder Center in Italy and Europe [41]. By further analyzing baseline and follow-up data, we expect to find specific clinical and instrumental markers for each domain assessed that can be helpful to the clinician in the diagnostic work-up and management of patient with parkinsonisms. Furthermore, our data could be used for comparison with other cohorts [6] and shared with national and international collaborators to develop new innovative projects. In addition, we could identify different patient populations with specific clinical characteristics that could be suitable for future studies assessing, for example, new and emerging MRI approaches like quantitative susceptibility mapping that might provide additional useful information for the early detection of PD and APs. We believe that our study will have important implications for clinical practice as well as in the research field.

## Abbreviations

PD: Parkinson’s disease
MSA: Multiple system atrophy
DLB: Dementia with Lewy bodies
PSP: Progressive supranuclear palsy
CBS: Corticobasal syndrome
uAP: Unspecified atypical parkinsonism
*N*: Number of patients
UPDRS-III: Unified PD Rating Scale – part III
LEDD: Levodopa equivalent daily dose
